# Seroprevalence of Measles-, Mumps-, and Rubella-specific antibodies in the German adult population – cross-sectional analysis of the German Health Interview and Examination Survey for Adults (DEGS1)

**DOI:** 10.1016/j.lanepe.2021.100128

**Published:** 2021-06-05

**Authors:** Nicole Friedrich, Christina Poethko-Müller, Ronny Kuhnert, Dorothea Matysiak-Klose, Judith Koch, Ole Wichmann, Sabine Santibanez, Annette Mankertz

**Affiliations:** a National Reference Centre Measles, Mumps, Rubella, Robert Koch Institute, Seestraße 10, 13353 Berlin, Germany; b Physical Health Unit, Robert Koch Institute, General-Pape-Straße 62-66, 12101 Berlin, Germany; c Epidemiological Data Centre, Robert Koch Institute, General-Pape-Straße 62-66, 12101 Berlin, Germany; d Immunization Unit, Robert Koch Institute, Seestraße 10, 13353 Berlin, Germany

## Abstract

**Background:**

The WHO European Region targets the elimination of measles, rubella, and the congenital rubella syndrome and welcomes mumps elimination via the joint MMR vaccine. In a push towards this elimination goal, Germany introduced a recommendation on MMR vaccination for adults in 2010 to prevent increasing numbers of measles cases among adults and to strengthen herd immunity.

**Methods:**

The prevalence of anti-measles, -mumps, and -rubella IgG antibodies was analysed in 7,115 participants between the ages of 18 and 79 years in the German Health Interview and Examination Survey. Risk factors of seronegativity of adults born 1970 or later were determined.

**Findings:**

The seroprevalence of anti-measles IgG antibodies was more than 97% in adults born before 1965 and less than 90% in adults born afterwards. Prevalence and GMTs declined with later years of birth. Seronegativity was associated with two-sided migration background and region of residence in East Germany. For anti-mumps IgG antibodies, the seroprevalence was less than 90% in almost all age groups. Prevalence and GMTs declined with later years of birth. Seronegativity was not associated with any socio-demographic factor. Anti-rubella IgG seropositivity was found in more than 90% of adults born before 1985. GMTs declined in younger age groups. Seronegativity was associated with birth between 1980 and 1993 and male gender. High socio-economic status lowered the odds of being seronegative.

**Interpretation:**

These data reinforce the implementation of the vaccination recommendation for adults and provide the basis for further evaluation of this measure.

**Funding:**

The Federal Ministry of Health, Germany.

**Research in context**

**Evidence before this study**

We did a literature search in PubMed for studies assessing the seroprevalence of anti-measles, -mumps, and -rubella antibodies published in English or German from database inception up to Jan 31, 2020. Key search terms included “measles”, “mumps”, or ”rubella” and “seroprevalence”, “serosurvey”, “IgG antibody”, “immunity”, or “cohort”. Most studies found focused on vaccination, analysed immunity of groups at special risk, or had limited representativity due to sample selection. However, evaluation of national vaccination strategies requires the analysis of data of population-based nation-wide serosurveys. Our research identified only one of such serosurveys based in Germany. This was KiGGS, the German national health survey on children and adolescents. No representative study assessing the seroprevalence of anti-measles, -mumps, and -rubella antibodies within the German adult population was identified.

**Added value of this study**

To our knowledge, this present analysis of DEGS1 data is the first representative study assessing the seroprevalence of anti-measles, -mumps, and -rubella IgG antibodies in the German adult population. Given the absence of a national vaccination registry, we therefore provide for the first time robust and representative data on immunity against those three diseases. Additionally, due to Germany´s history of division and reunification, we were able to compare the influence of two distinct vaccination strategies on a population.

**Implications of all the available evidence**

Data of this study provide basis for the evaluation of national vaccination policies and may also be used by other European countries with similar disease epidemiology in order assess their vaccination strategies. Our data emphasize the presence of an instable transition phase between natural herd immunity of a population and herd immunity acquired by vaccination which renders a population susceptible to virus circulation irrespective of whether a country implemented compulsory vaccination or a recommendation to vaccinate.

Alt-text: Unlabelled box

## 1 Introduction

Measles, mumps, and rubella are highly contagious viral diseases that are still of major public health concern despite the availability of safe and effective vaccines. In 2018, an estimate of more than 140,000 individuals died from measles globally [1]. Virus-induced transient immunosuppression increases the susceptibility to infections especially within the first year after diagnosis [2]. Often observed are bacterial superinfections causing otitis media, bronchitis, pneumonia, and diarrhea. Further complications are acute post-infectious encephalitis and subacute sclerosing panencephalitis (SSPE), a slowly progressing measles infection of the central nervous system [3]. Mumps is characterised by parotitis and fever. Mumps virus is neurotropic with involvement of the central nervous system in half of the cases, where it can induce aseptic meningitis or encephalitis. Other complications include orchitis, pancreatitis, and deafness [4]. Rubella is usually a mild acute disease. However, rubella infection during pregnancy may result in miscarriage, foetal death, or congenital defects of the foetus called congenital rubella syndrome (CRS) [5]. About 105,000 children with CRS are estimated to be born globally each year [6].

The WHO targets the elimination of measles, rubella, and CRS in the European region [7]. Accordingly, Germany aims achieving and maintaining population immunity at levels that prevent virus transmission. Under consideration of the respective reproductive number R_0_, population immunity to prevent virus transmission is estimated to be 92–94% for measles (R_0_=12–18) and 83–85% for rubella (R_0_=5–8) [8].

History of measles, mumps, rubella (MMR) vaccination differs between East and West Germany (supplementary fig. S1). In East Germany, measles vaccination became obligatory for 8 months old children in 1970. In 1983, a second vaccination against measles was recommended for children aged between 2 and 16 years, who were vaccinated before 1 year of age. From 1986 to 1990 (reunification), two vaccinations against measles were compulsory [9,10]. West Germany implemented a recommendation in 1974 [11]. A general recommendation of vaccination against mumps was never introduced in East Germany and only available after reunification in 1991. In West Germany, mumps vaccination was recommended from 1976 onwards [10,12]. The rubella vaccine was not generally available in East Germany. In West Germany, rubella vaccination was recommended selectively to girls since 1976 and to all children since 1981 [13]. In reunited Germany, a two-fold MMR vaccination is suggested to every child since 1991 [14]. The current recommendation of the German Standing Committee on Vaccination (STIKO) is to vaccinate children at the age of 11 months and to apply a second dose at the age of 15 months [15]. In addition, a recommendation to vaccinate adults born after 1970 who have never been vaccinated, had only one vaccination during childhood or have an unclear vaccination status was implemented in 2010 [16]. Women of childbearing age should be vaccinated twice against rubella [15]. Since March 2020, a two-fold measles vaccination is according to law statutory for children when entering care facilities or schools and for adults born after 1970 working in medical or community facilities unless immunity can be proven otherwise (Measles Protection Act) [17].

Serosurveys are a powerful tool to assess population immunity and national vaccination policies. The German Health Interview and Examination Survey for Adults (DEGS1) is part of Germany´s health monitoring. Prior to DEGS1, no representative sero-epidemiological data on the prevalence of anti-measles, -mumps, and -rubella antibodies for adults in Germany was available. Aim of this study was to describe MMR seroprevalence in the German adult population before the implementation of the vaccination recommendation for adults thereby serving as a basis for the evaluation of this measure and to identify predictors for seronegativity of anti-measles, -mumps, and -rubella antibodies in the era of vaccination.

## 2 Methods

**Survey design and data collection.** The German Health Interview and Examination Survey for Adults (DEGS1) is part of the health monitoring conducted by the Robert Koch-Institute. Design and methodical details of DEGS1, including details on statistical power and sample size calculations, have been described elsewhere [18,19]. Briefly, DEGS1 is an interview and examination survey of the German adult population aged 18 years and older and was conducted between November 2008 and December 2011. The DEGS1 study population is composed out of former participants of the German National Health Interview and Examination Survey 1998 (GNHIES98) and newly invited DEGS1 participants (supplementary fig. S2). GNHIES98 participants who agreed to be re-contacted and were still contactable were re-invited to take part in DEGS1. In order to preserve representativeness, n = 11,008 participants were newly invited to participate. Those were chosen based on two-stage stratified cluster sampling [20]. For this, in a first step, 180 primary sampling units were determined from a list of all German municipal communities stratified according to districts, and a classification system that takes into account the grade of urbanization, regional population density, and administrative borders. In a second step, random samples of individuals stratified by 10-year age-groups were selected from local population registries [18]. In total, 8,151 participants took part, with a response rate of 42% of first-invited and 62% of re-invited participants [19]. Cross-sectional analyses were performed on participants aged 18–79 years with examination and interview data available of 7,115 participants [18]. Antibody titres were determined of 6,802 (measles), 6,790 (mumps), and 6,811 (rubella) participants, respectively (supplementary fig. S2). Information on socio-economic status (SES) was lacking of 48 participants and on migration background on 232 participants, respectively. Titres were not determined of all participants due to technical reasons. Missing analysis revealed that the population of analysis was not statistically significantly affected by missing titres with respect to gender, year of birth, region of residence, SES or migration background. Variables relevant for the present analysis (MMR IgG titres, year of birth, region of residence, migration background, SES and gender) were collected by questionnaires (self-administered or computer-assisted personal interview) [20,18], except for MMR IgG titres which were determined by ELISA of serum samples. SES was determined using an index including information on education, net household income, professional status, employment history, and retirement which allowed classification into low, middle, or high SES groups [21].

**MMR IgG ELISA.** Anti-MMR IgG titres were determined by anti-measles virus-ELISA (IgG), anti-mumps virus-AT-ELISA (IgG), and anti-rubella virus-ELISA (IgG) (all from Euroimmun) using an automated processor (Analyzer I-4P; Euroimmun). Titres were calculated by correlation of the ELISA results to a standard curve using control sera (measles: calibrated to the 3rd International Standard NIBSC 97/648) and interpreted according to the manufacturer´s instructions. Measles: negative: <200 IU/l, equivocal: ≥200 and <275 IU/l, positive: ≥275 IU/l, lower detection limit: 8 IU/l, upper detection limit: 5,000 IU/l. Mumps: negative: <16 RU/ml, equivocal: ≥16 and <22 RU/ml, positive: ≥22 RU/ml, lower detection limit: 0⋅5 RU/ml, upper detection limit: 200 RU/ml. Rubella: negative: <8 IU/ml, equivocal: ≥8 and <11 IU/ml, positive: ≥11 IU/ml, lower detection limit: 0⋅3 IU/ml, upper detection limit: 200 IU/ml.

**Statistical analysis.** Data were analysed with Stata/SE version 15.1 (StataCorp, Texas, USA) using survey procedures for complex samples allowing to adequately account for the clustering of participants within one sampling unit and to apply weighting factors throughout the analyses in order to correct deviations in the sample from the German general population regarding age, gender, region of residence, nationality, community class, level of education (reference date: 31st December 2010), and re-participation rate of former GNHIES98 participants, and thus to obtain estimates representative at national level [19]. Dichotomisations were performed by combining positive and equivocal titres in the positive category. Multivariate odds ratios were determined for participants born 1970 or later by multivariate binary logistic regression with serostatus of anti-measles, -mumps, or -rubella IgG antibodies (negative/not negative) as the dependent variable and the socio-demographic factors year of birth (10-year age groups), region of residence (West/East Germany incl. Berlin), migration background (none/one-/two-sided), SES (low/middle/high) and gender (men/women) as independent variables. A p-value <0⋅05 was considered to be statistically significant. To calculate geometric mean titres (GMTs), MMR IgG titres beyond the detection limit of the respective ELISA system were modelled. Imputation was conducted separately for values above and below the ELISA detection limit and performed stratified by age (year of birth ≤1960 and >1960). Modeling of values above the detection limit was performed by including normally distributed values only (measles: ≥1,000 IU/l, rubella: ≥50 IU/ml, mumps: all values). Since ELISA values are ≥0, values in the lower measuring range were transformed to logarithmic scale for imputation. Since transformation did not result in normally distributed values, imputation of values beyond the lower detection limit was performed using a step function.

### 2.1 Ethics approval and reporting

The study was approved by the ethics committee of the Charité University Medicine Berlin, Germany, and the Federal Office for the Protection of Data. DEGS1 conforms to the Declaration of Helsinki. Participants provided written informed consent prior to the interview and examination. The present report complies with the STROBE guidelines for observational studies [22].

**Role of funding source.** The funder had no role in study design, data collection, data analysis, interpretation, or writing of the report. All authors had full access to all the data in the study and had final responsibility for the decision to submit for publication.

## 3 Results

### 3.1 Seroprevalence of MMR antibodies

Seroprevalence of MMR IgG antibody titres in the German adult population aged 18–79 years is shown in table 1. A description of characteristics of the analysed study participants is shown in supplementary table S1.

**Table 1:** Seroprevalences of measles, mumps, and rubella IgG antibody titres in the German adult population by socio-demographic factors in 2008–2011. (^a^measles: *n* = 6,802, mumps: *n* = 6,790, rubella: *n* = 6,811; ^b^measles: *n* = 6,757, mumps: *n* = 6,745, rubella: *n* = 6,766).

	Measles Titre			Mumps Titre			Rubella Titre		
	Negative% (95% CI)	Equivocal% (95% CI)	Positive% (95% CI)	Negative% (95% CI)	Equivocal% (95% CI)	Positive% (95% CI)	Negative% (95% CI)	Equivocal% (95% CI)	Positive% (95% CI)
Total^a^	6⋅4 (5⋅6–7⋅3)	3⋅7 (3⋅0–4⋅5)	89⋅9 (88⋅6–91⋅1)	10⋅3 (9⋅3–11⋅3)	5⋅5 (4⋅8–6⋅3)	84⋅2 (82⋅9–85⋅4)	4⋅4 (3⋅8–5⋅1)	1⋅6 (1⋅3–2⋅0)	94⋅0 (93⋅3–94⋅7)
Gender^a^									
Men	6⋅2 (5⋅1–7⋅6)	4⋅5 (3⋅5–5⋅6)	89⋅3 (87⋅4–91⋅0)	10⋅0 (8⋅9–11⋅3)	6⋅4 (5⋅3–7⋅6)	83⋅6 (81⋅9–85⋅2)	5⋅7 (4⋅8–6⋅8)	1⋅4 (0⋅9–2⋅0)	93⋅0 (91⋅8–94⋅0)
Women	6⋅6 (5⋅4–7⋅9)	2⋅9 (2⋅3–3⋅8)	90⋅5 (89⋅1–91⋅8)	10⋅5 (9⋅2–12⋅1)	4⋅7 (3⋅9–5⋅7)	84⋅8 (83⋅1–86⋅4)	3⋅1 (2⋅4–4⋅0)	1⋅8 (1⋅3–2⋅4)	95⋅1 (94⋅1–96⋅0)
Year of birth^a^									
1928–1934	0⋅9 (0⋅1–6⋅3)	0⋅1 (0⋅0–1⋅0)	99⋅0 (94⋅3–99⋅8)	2⋅2 (1⋅0–4⋅6)	3⋅0 (1⋅7–5⋅4)	94⋅8 (91⋅9–96⋅7)	1⋅7 (0⋅7–4⋅2)	3⋅5 (1⋅3–9⋅0)	94⋅8 (89⋅3–97⋅6)
1935–1939	0⋅6 (0⋅1–3⋅6)	0⋅4 (0⋅1–1⋅7)	99⋅0 (96⋅6–99⋅7)	6⋅2 (4⋅3–8⋅9)	4⋅3 (2⋅6–7⋅0)	89⋅5 (85⋅8–92⋅3)	3⋅8 (2⋅4–5⋅9)	1⋅7 (1⋅0–3⋅1)	94⋅5 (92⋅0–96⋅3)
1940–1944	1⋅0 (0⋅4–2⋅2)	0⋅2 (0⋅1–0⋅4)	98⋅9 (97⋅7–99⋅5)	3⋅9 (2⋅6–5⋅8)	4⋅2 (2⋅8–6⋅2)	91⋅9 (89⋅5–93⋅8)	2⋅9 (1⋅6–5⋅3)	1⋅7 (0⋅8–3⋅5)	95⋅4 (92⋅6–97⋅2)
1945–1949	0⋅2 (0⋅0–0⋅7)	1⋅4 (0⋅5–3⋅5)	98⋅5 (96⋅4–99⋅4)	7⋅7 (5⋅2–11⋅4)	3⋅3 (1⋅8–6⋅0)	89⋅0 (85⋅0–92⋅1)	4⋅5 (2⋅6–7⋅7)	1⋅8 (0⋅7–4⋅5)	93⋅7 (90⋅2–96⋅0)
1950–1954	0⋅3 (0⋅1–1⋅0)	0⋅8 (0⋅3–2⋅2)	98⋅9 (97⋅5–99⋅5)	7⋅6 (5⋅4–10⋅5)	3⋅4 (2⋅1–5⋅3)	89⋅1 (85⋅7–91⋅7)	2⋅8 (1⋅7–4⋅6)	0⋅9 (0⋅3–2⋅5)	96⋅3 (94⋅2–97⋅6)
1955–1959	0⋅7 (0⋅3–1⋅6)	0⋅6 (0⋅2–1⋅7)	98⋅7 (97⋅5–99⋅3)	8⋅0 (5⋅9–10⋅9)	6⋅5 (4⋅5–9⋅1)	85⋅5 (82⋅0–88⋅4)	1⋅4 (0⋅8–2⋅6)	1⋅2 (0⋅6–2⋅5)	97⋅4 (95⋅8–98⋅4)
1960–1964	1⋅2 (0⋅6–2⋅3)	1⋅6 (0⋅7–3⋅6)	97⋅2 (95⋅2–98⋅4)	10⋅0 (7⋅3–13⋅4)	4⋅7 (3⋅1–7⋅0)	85⋅4 (81⋅7–88⋅4)	3⋅1 (1⋅8–5⋅6)	0⋅3 (0⋅1–0⋅8)	96⋅6 (94⋅2–98⋅0)
1965–1969	8⋅1 (5⋅6–11⋅5)	3⋅0 (1⋅6–5⋅4)	89⋅0 (85⋅2–91⋅9)	12⋅9 (9⋅8–16⋅9)	4⋅3 (2⋅6–7⋅1)	82⋅8 (78⋅2–86⋅6)	4⋅4 (2⋅6–7⋅6)	0⋅7 (0⋅3–1⋅6)	94⋅9 (91⋅7–96⋅9)
1970–1974	12⋅8 (9⋅5–17⋅1)	5⋅2 (3⋅3–8⋅0)	82⋅0 (76⋅5–86⋅5)	11⋅1 (8⋅5–14⋅4)	6⋅4 (4⋅1–9⋅9)	82⋅5 (78⋅3–86⋅0)	3⋅7 (2⋅1–6⋅5)	0⋅8 (0⋅2–2⋅6)	95⋅5 (92⋅7–97⋅3)
1975–1979	16⋅2 (11⋅6–22⋅2)	8⋅0 (5⋅1–12⋅2)	75⋅8 (69⋅5–81⋅3)	19⋅8 (14⋅8–25⋅8)	8⋅5 (5⋅8–12⋅2)	71⋅8 (65⋅6–77⋅3)	5⋅4 (2⋅9–9⋅9)	1⋅1 (0⋅3–4⋅2)	93⋅5 (88⋅9–96⋅3)
1980–1984	17⋅5 (13⋅0–23⋅1)	8⋅7 (5⋅9–12⋅6)	73⋅9 (67⋅5–79⋅3)	15⋅1 (11⋅7–19⋅2)	9⋅9 (6⋅8–14⋅3)	75⋅0 (69⋅7–79⋅7)	6⋅7 (4⋅3–10⋅1)	1⋅6 (0⋅6–4⋅3)	91⋅8 (87⋅9–94⋅5)
1985–1993	14⋅5 (11⋅6–17⋅9)	11⋅1 (8⋅4–14⋅4)	74⋅4 (70⋅2–78⋅3)	13⋅7 (11⋅0–17⋅0)	6⋅9 (5⋅1–9⋅4)	79⋅4 (75⋅8–82⋅6)	9⋅6 (7⋅5–12⋅3)	4⋅3 (3⋅0–6⋅1)	86⋅1 (83⋅1–88⋅7)
Region of residence^a^									
West	5⋅6 (4⋅8–6⋅5)	3⋅0 (2⋅3–3⋅8)	91⋅5 (90⋅2–92⋅7)	10⋅7 (9⋅7–11⋅9)	5⋅6 (4⋅8–6⋅6)	83⋅7 (82⋅2–85⋅1)	4⋅6 (3⋅9–5⋅4)	1⋅6 (1⋅2–2⋅0)	93⋅9 (93⋅0–94⋅7)
East	9⋅5 (7⋅6–11⋅8)	6⋅3 (5⋅1–7⋅9)	84⋅2 (81⋅3–86⋅6)	8⋅6 (7⋅0–10⋅4)	5⋅3 (4⋅2–6⋅8)	86⋅1 (83⋅7–88⋅2)	3⋅7 (2⋅8–4⋅8)	1⋅7 (1⋅1–2⋅5)	94⋅7 (93⋅3–95⋅8)
Socio-economic status^b^									
Low	7⋅8 (5⋅8–10⋅4)	3⋅3 (2⋅2–5⋅0)	88⋅9 (86⋅0–91⋅2)	12⋅3 (10⋅0–14⋅9)	6⋅7 (5⋅0–8⋅9)	81⋅1 (78⋅1–83⋅7)	5⋅5 (3⋅9–7⋅6)	1⋅9 (1⋅2–3⋅2)	92⋅6 (90⋅4–94⋅3)
Middle	6⋅2 (5⋅2–7⋅4)	3⋅7 (3⋅0–4⋅6)	90⋅1 (88⋅5–91⋅4)	8⋅9 (7⋅8–10⋅2)	5⋅2 (4⋅4–6⋅1)	85⋅9 (84⋅4–87⋅4)	4⋅0 (3⋅3–4⋅9)	1⋅6 (1⋅2–2⋅2)	94⋅3 (93⋅3–95⋅2)
High	5⋅6 (4⋅2–7⋅5)	3⋅6 (2⋅3–5⋅6)	90⋅8 (88⋅5–92⋅7)	12⋅3 (10⋅3–14⋅6)	5⋅7 (4⋅4–7⋅5)	82⋅0 (79⋅4–84⋅3)	4⋅4 (3⋅4–5⋅8)	1⋅1 (0⋅6–1⋅7)	94⋅6 (93⋅1–95⋅7)

Anti-measles IgG was found in 89⋅9% (95% CI 88⋅6-91⋅1) of German adults, 3⋅7% (95% CI 3⋅0-4⋅5) had an equivocal titre and 6⋅4% (95% CI 5⋅6-7⋅3) were seronegative. While there were hardly any gender- or SES-specific differences, seroprevalence clearly differed dependent on year of birth. More than 97% of adults born before 1965, but only 89% (95% CI 85⋅2-91⋅9%) of adults born between 1965 and 1969 were seropositive for anti-measles IgG. This proportion further declined with later years of birth. Only slightly more than 80% of adults born between 1970 and 1974 and clearly less than 80% of adults born 1975 or later were seropositive for anti-measles IgG. This analysis revealed a turning point in seropositivity among adults born between 1960 and 1964 and adults born between 1965 and 1969. The decrease in seropositivity correlated with an increase of both, sera with equivocal and with negative titres. Interestingly, adults, resident in West Germany, were more often seropositive for anti-measles IgG than adults living in East Germany (91⋅5% (95% CI 90⋅2–92⋅7) and 84⋅2% (95% CI 81⋅3–86⋅6), respectively) (table 1).

**Fig. 1:**
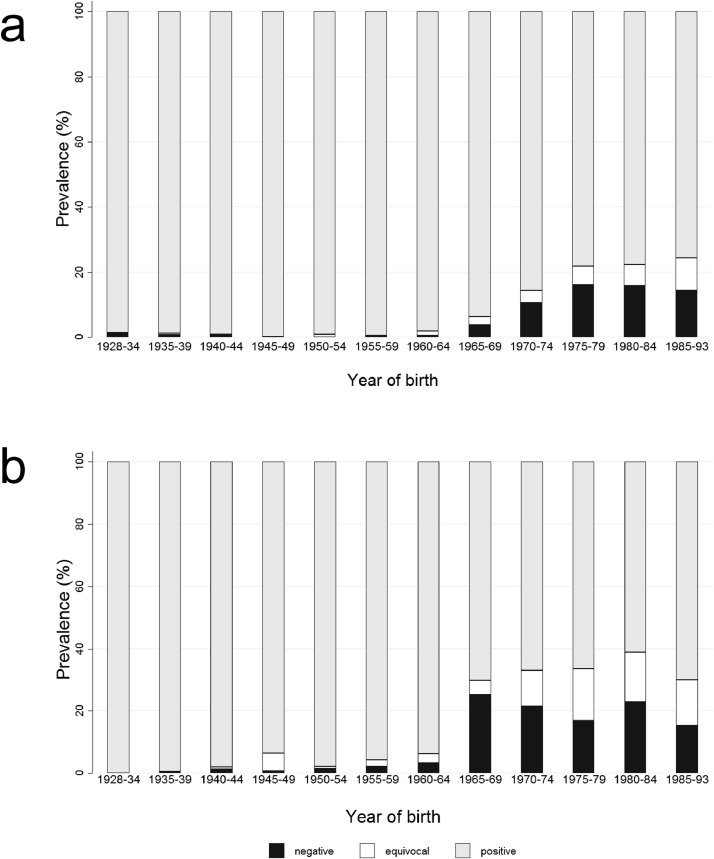
Distribution of seroprevalence of measles IgG antibody titres (%) in the German adult population in (a) West Germany and (b) East Germany dependent on their year of birth in 2008–2011 (*n* = 6,802).

When seroprevalence was analysed dependent on both, region of residence and year of birth, the group of adults born between 1965 and 1969 exhibited the most prominent difference. As shown in Fig. 1, there was a sharp increase of anti-measles IgG seronegative adults resident in East Germany in this age group (Fig. 1b). In contrast, the proportion of seronegative adults resident in West Germany rose only slightly (Fig. 1a). Also, adults in East Germany born 1965 or later had more often equivocal anti-measles IgG titres than their counterparts in West Germany (Fig. 1).

Seropositivity of anti-mumps IgG of German adults was overall 84⋅2% (95% CI 82⋅9-85⋅4), 10⋅3% (95% CI 9⋅3-11⋅3) were seronegative and 5⋅5% (95% CI 4⋅8-6⋅3) displayed equivocal titres. More than 89% of adults born between 1928 and 1954 were seropositive. The share of seropositive adults decreased with later years of birth. Least seropositive were adults born between 1975 and 1979 (71⋅8% (95% CI 65⋅6-77⋅3)). Hardly any differences in seroprevalence were observed dependent on gender, region of residence, or SES.

**Fig. 2:**
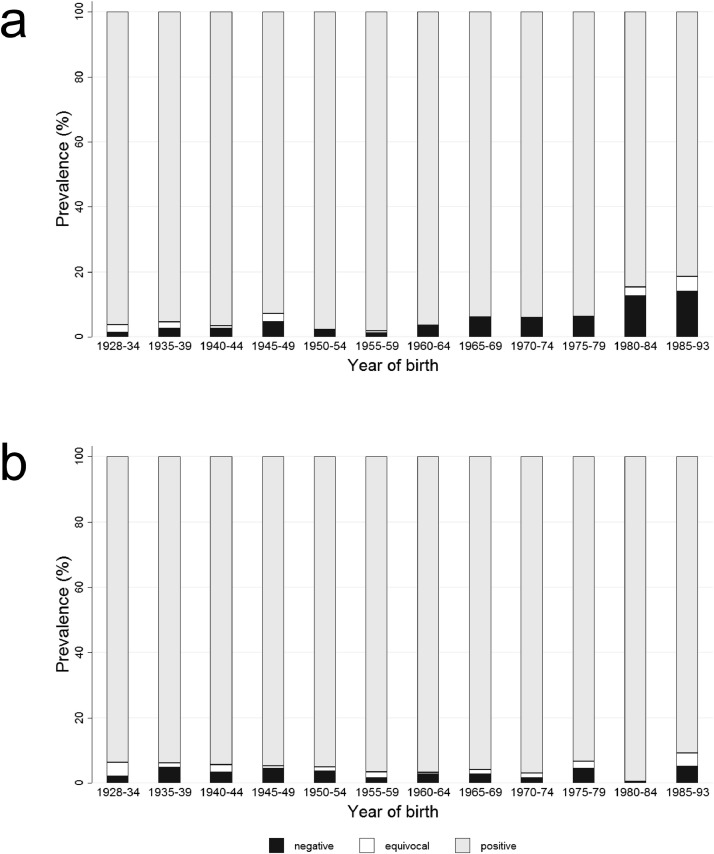
Age-specific distribution of seroprevalence of rubella IgG antibody titres (%) in the German adult population of (a) men and (b) women in 2008–2011 (*n* = 6,811).

With regards to anti-rubella IgG, in total, 94⋅0% (95% CI 93⋅3-94⋅7) of adults were seropositive, 4⋅4% (95% CI 3⋅8-5⋅1) seronegative and 1⋅6% (95% CI 1⋅3-2⋅0) had an equivocal titre. There were no obvious differences in seropositivity dependent on gender, region of residence, and SES. Remarkably, more than 90% of adults born between 1928 and 1984 were seropositive. This proportion decreased to 86⋅1% (95% CI 83⋅1-88⋅7) among adults born between 1985 and 1993. Analysis of seroprevalence dependent on both, gender and year of birth, revealed men born between 1980 and 1993 being clearly less seropositive than women born at the same time (Fig. 2a). Women born between 1980 and 1984 were nearly 100% seropositive (99⋅39% (95% CI 98⋅08–99⋅81)). This proportion decreased among women born between 1985 and 1993 (90⋅84% (95% CI 87⋅16–93⋅54)) (Fig. 2b).

### 3.2 Predictors of negative MMR antibody titres

Multivariate odds ratios for the association between socio-demographic factors and negative MMR antibody titres were determined for German adults born 1970 or later (for unadjusted odds ratios see supplementary table S2). This analysis proved the significant association of anti-measles IgG seroprevalence and region of residence. Adults resident in East Germany were more likely seronegative against measles than adults resident in West Germany (OR 1⋅6; 95% CI 1⋅08-2⋅37) (table 2). Also migration background significantly influenced the risk for negative anti-measles titres. Adults with a two-sided migration background were more than two times more likely seronegative than adults with no migration background (OR 2⋅35; 95% CI 1⋅56-3⋅54). Gender, year of birth or SES did not significantly influence anti-measles seroprevalence.

**Table 2:** Multivariate odds ratios (OR) for the association between socio-demographic factors and negative measles, mumps, and rubella antibody titres in German adults born 1970 or later in 2008–2011 (measles: *n* = 1,845, mumps: *n* = 1,570, rubella: *n* = 1,723).

	Measles		Mumps		Rubella	
	OR (95% CI)	p	OR (95% CI)	p	OR (95% CI)	p
Gender		1⋅00		0⋅25		<0⋅01
Men	1⋅00 (0⋅70–1⋅43)		0⋅83 (0⋅61–1⋅13)		3⋅74 (2⋅12–6⋅58)	
Women	Referent		Referent		Referent	
Year of birth		0⋅71		0⋅65		0⋅01
1970–1979	Referent		Referent		Referent	
1980–1993	1⋅07 (0⋅75–1⋅53)		0⋅93 (0⋅68–1⋅27)		1⋅79 (1⋅18–2⋅71)	
Region of residence		0⋅02		0⋅42		0⋅03
West	Referent		Referent		Referent	
East	1⋅60 (1⋅08–2⋅37)		0⋅86 (0⋅60–1⋅24)		0⋅54 (0⋅31–0⋅94)	
Socio-economic status		0⋅96		0⋅37		0⋅07
Low	Referent		Referent		Referent	
Middle	0⋅95 (0⋅62–1⋅44)		0⋅78 (0⋅53–1⋅14)		0⋅61 (0⋅37–1⋅02)	
High	0⋅99 (0⋅58–1⋅68)		0⋅93 (0⋅59–1⋅47)		0⋅47 (0⋅23–0⋅95)	
Migration background		<0⋅01		0⋅59		0⋅07
None	Referent		Referent		Referent	
One-sided	0⋅78 (0⋅37–1⋅65)		0⋅88 (0⋅42–1⋅84)		1⋅81 (0⋅94–3⋅48)	
Two-sided	2⋅35 (1⋅56–3⋅54)		1⋅16 (0⋅76–1⋅77)		0⋅66 (0⋅34–1⋅28)	

Anti-mumps IgG seronegativity was not significantly associated with any socio-demographic factor tested.

Seroprevalence of anti-rubella IgG was significantly associated with gender and year of birth: the risk of being seronegative was more than three times higher for men than for women (OR 3⋅74; 95% CI 2⋅12-6⋅58), and almost two times higher for adults born between 1980 and 1993 than for adults born between 1970 and 1979 (OR 1⋅79; 95% CI 1⋅18-2⋅71) (table 2). Adults resident in East Germany had lower odds of being seronegative than their counterparts in West Germany (OR 0⋅54; 95% CI 0⋅31-0⋅94). High SES resulted in lower odds of having a negative titre (OR 0⋅47; 95% CI 0⋅23-0⋅95) while migration background was not significantly associated with seroprevalence of anti-rubella antibodies.

### 3.3 MMR antibody level over time

**Fig. 3:**
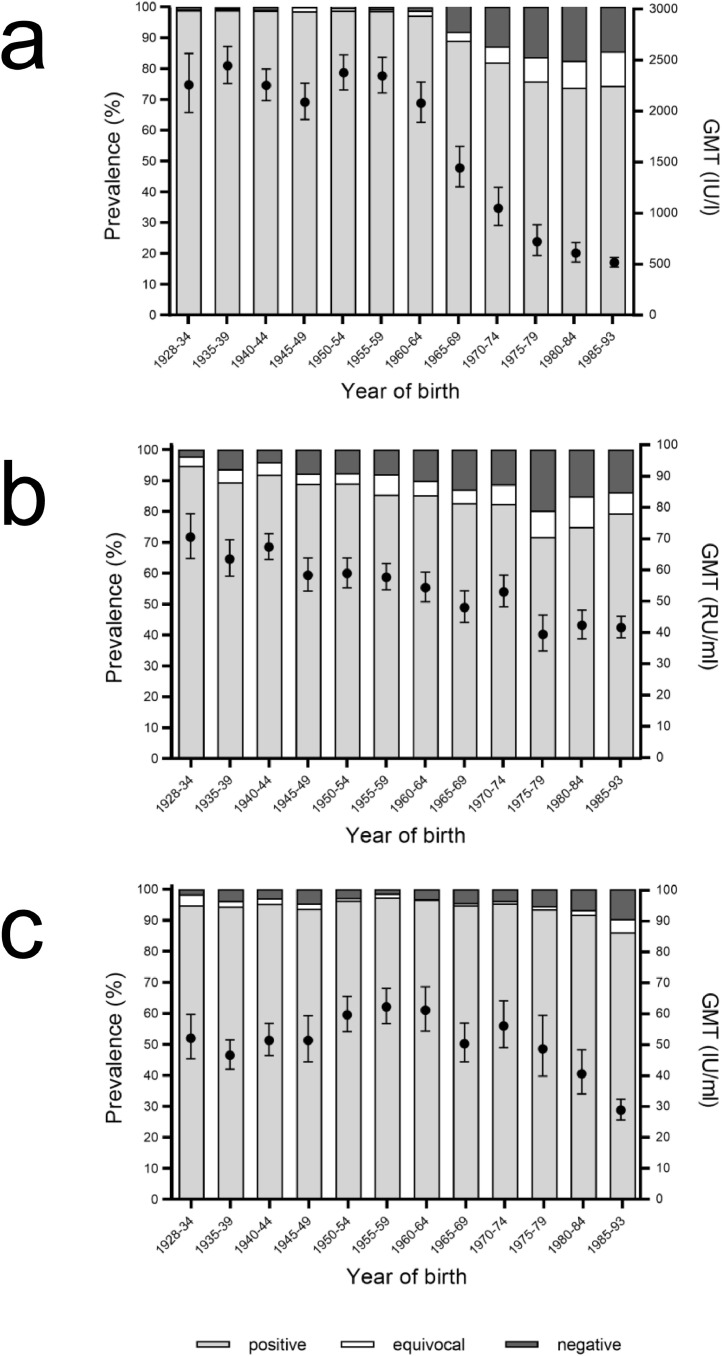
Distribution of prevalence of (a) measles, (b) mumps, and (c) rubella antibodies (%) (columns) and GMTs (circles) in the German adult population dependent on their year of birth in 2008–2011 (measles: *n* = 6,802, mumps: *n* = 6,790, rubella: *n* = 6,811).

The distribution of anti-MMR IgG GMTs and seroprevalence was analysed with respect to year of birth. Anti-measles GMTs were higher than 2,000 IU/l in adults born before 1965 (Fig. 3a). GMTs started to decline to 1,443⋅50 IU/l (95% CI 1,257⋅76–1,656⋅67) in adults born between 1965 and 1969, paralleling the decrease of seroprevalence of anti-measles IgG. The descent of GMT and seropositivity continued with later years of birth, with a GMT being around 500 IU/l in the youngest age group born between 1985 and 1993.

As shown in Fig. 3b, also anti-mumps GMTs decreased with later years of birth, even though the effect was not as pronounced as observed for measles. A decrease of anti-rubella GMTs among the younger age groups was also observed (Fig. 3c). While anti-rubella GMTs of adults born between 1928 and 1964 ranged between 46⋅65 IU/ml and 62⋅28 IU/ml (95% CI 42⋅16–51⋅61 and 56⋅84-68⋅25, respectively), GMTs of adults born between 1975 and 1993 declined continuously to the youngest age group (28⋅85 IU/ml (95% CI 25⋅71-32⋅37)).

## 4 Discussion

### 4.1 Main results

The seroprevalence of anti-measles IgG antibodies was more than 97% in adults born before 1965. Adults born later were 73⋅9–89⋅0% seropositive. Prevalence as well as GMTs declined with later years of birth. Seronegativity of adults born 1970 or later was significantly associated with two-sided migration background and a region of residence in East Germany.

For anti-mumps IgG antibodies, the seroprevalence was less than 90% in all age groups apart from the elderly born between 1928 and 1934 or between 1940 and 1944. Prevalence and GMTs declined with later years of birth but the effect was less pronounced compared to measles. Anti-mumps IgG seronegativity of adults born 1970 or later was not significantly associated with any socio-demographic factor.

Anti-rubella IgG seropositivity was found in more than 90% of adults born before 1985. GMTs declined in the younger age groups born 1975 or later. Anti-rubella IgG seronegativity of adults born 1970 or later was significantly associated with birth between 1980 and 1993 and male gender. High SES lowered the risk of being seronegative.

### 4.2 Measles

Anti-measles vaccination strategies differed between East and West Germany. Accordingly, we observed regional differences in the prevalence of anti-measles IgG. The proportion of seropositive adults in East Germany was lower than in West Germany. Due to the obligation to vaccinate against measles, vaccination rates in East Germany were high; generally around 92%, in parts of the population 60–70% [10]. The rapid increase of the proportion of seronegative East Germans born between 1965 and 1969 may therefore be due to non-responders to vaccination and/or less natural measles infections among susceptible individuals due to the reduction of virus circulation and only rare imports of measles (supplementary fig. S3). Moreover, seroconversion rates have been shown to be lower when the vaccine is applied during the first year of life compared to children having received the vaccine later in life [23]. The policy of East Germany to vaccinate children already at the age of 8 months therefore may have added to this effect [10]. The initial low thermostability of the vaccine applied in East Germany may have also resulted in less seroconversion [24]. By contrast, vaccination rates in West Germany were generally clearly lower still allowing for natural infections and the respective seroconversion resulting in a more slowly increase of seronegatives over years of birth [25]. Fittingly, also other European countries observed sharp increases in seronegativity after introduction of mandatory vaccination while the increase was only moderate in countries with voluntary vaccination [26], [27], [28], [29].

Anti-measles seronegativity was especially high in younger adults with two-sided migration background. Those possibly involve immigrants who made use of the vaccination program of their home countries. Inadequate execution of the program or factors influencing measles vaccine efficacy such as cold chain interruption or higher UV indices may have resulted in less seroconversion [30]. Population-specific humoral immune responses may contribute to the observed association [31].

Several studies observed lower anti-measles IgG titres and waning humoral immunity after vaccination in contrast to natural measles infections before [32], [33], [34], [35], [36], [37]. Vaccinated individuals whose titres are classified as equivocal or negative often have sufficient anti-measles antibodies with neutralizing capacity [23]. However, if population immunity remains below the level needed for herd immunity, waning humoral immunity may pose a major problem for children in their first year of life. Lower levels of maternal antibodies of vaccinated mothers may lead to a gap of protection until the children can get vaccinated. Unfortunately, measles infections in the first year of life are commonly associated with complications and the late sequela SSPE [3].

A population immunity of 92–94% is estimated to be required for measles elimination [8]. Only German adults born before 1965 fulfilled this condition. Our data thus support the implementation of the vaccination recommendation for adults by the STIKO as well as of the Measles Protection Act but also suggest that a considerable proportion of adults born between 1965 and 1969 would benefit from measles vaccination [38]. However, the benefit of inclusion of this age group in the vaccination recommendation would be opposed by vaccination of a high number of already immune adults.

### 4.3 Mumps

Mumps outbreaks have been observed in Germany and other European countries as well as in the USA and Canada, all countries demonstrating high vaccination coverage. Mainly affected were young adults in college and university settings with a history of remote vaccination [39], [40], [41]. Similar to what has been noted before, we found lower GMTs in response to vaccination [42]. This in combination with waning immunity and the observed low seroprevalence may be an explanation for the observed outbreaks [32,34,37]. Regional clusters of insufficiently vaccinated individuals may additionally facilitate mumps outbreaks [43].

Differences in vaccination policies between East and West Germany did not come into effect. Presumably, low vaccination rates warranted a high number of natural mumps virus infections also in West Germany [25], obliterating a possible effect of vaccination-related factors on mumps immunity.

Least seropositive were adults born between 1975 and 1979 who were probably particularly affected by poor realisations of vaccination recommendations along with reduced virus circulation. A third vaccination may be considered to booster anti-mumps immunity in the adult population or be applied in the event of an outbreak similar to the USA [44,45].

### 4.4 Rubella

The implementation of rubella vaccination in West Germany resulted in less seropositive adults compared to East Germany. Reduced virus circulation due to vaccinations may have diminished the occurrence of natural rubella infections in West Germany in younger age-groups, similar to what we suppose for measles in East Germany.

Rubella seronegativity in German children and adolescents was described to be associated with high levels of maternal education and a resulting decision against vaccination [36,46]. By contrast, we observed younger adults of high SES more likely to be seropositive than adults of low SES. Presuming the effect to be vaccination-related, this discrepant observation may reside in the fact that the variable SES relates to the participant and not to the parent who decided on vaccination and may differ in the level of education. Moreover, time-dependent differences in media usage between parents of DEGS1 participants and parents of the aforementioned children and adolescents may have diversely affected their decision on vaccination. In addition, female DEGS1 participants may have decided to get vaccinated during family planning. Women pro-actively closing immunization gaps with individual benefit may most probably be of high SES.

The West German recommendation to vaccinate against rubella initially focused on girls. Although it was extended to all children in their second year of life in 1981, the explicit recommendation to vaccinate adolescent girls remained until 1998. East Germany adopted this policy after reunification in 1991 [[10], [11], [12], [13], [14],^47^]. This resulted in a higher proportion of seronegative men as also observed in other European countries with initial selective vaccination of girls and opposed to countries with mandatory vaccination for all children [48], [49], [50], [51]. Those seronegative men pose a risk to unprotected women. This is of particular importance since there is a sharp increase of negative IgG titres in women of childbearing age which may be a result of the omission of the additional vaccination in adolescence. Both risk groups are considered for anti-rubella catch-up vaccination strategies by STIKO. Lower levels of anti-rubella IgG in response to the introduction of vaccination as well as waning immunity have been observed before [32], [33], [34], [35], [36], [37]. Still, antibodies of high avidity most probably provide excellent protection even if present in only low amounts. To prevent rubella transmission, the WHO asks for a population immunity of 83–85% which was reached in all age groups. Accordingly, there was no indication of endemic circulation of rubella in Germany in 2019 and Germany was declared free from endemic rubella while this study was under revision [52,53].

### 4.5 Strength and weaknesses

Seroprevalence studies on anti-measles, -mumps, and -rubella IgG antibodies in the German population are rare and target mainly groups of special interest [54,55]. So far, only the KiGGS study collected data representative for the whole of Germany, however of children and adolescents [36]. Thus, DEGS1 is the first nationally representative survey of the German adult population providing reliable data on anti-measles, -mumps, and -rubella IgG. Thereby, DEGS1 allows the evaluation of national vaccination strategies which is of particular importance since data on vaccination of German adults is incomplete.

The sustainable reduction of measles cases and severe measles complications as well as efforts to eliminate measles is a joint European if not global task. Globalization, free travel and migration, especially within the European region, facilitate virus spread among countries as long as no sufficient herd immunity is present within the entire region. Our data are in line with other European studies which discovered a decline of IgG seroprevalence among adults in relation with the introduction of anti-measles vaccination [[26], [27], [28], [29],[56], [57], [58]]. This emphasizes the presence of an instable transition phase between natural herd immunity of a population and herd immunity acquired by vaccination which renders a population susceptible to virus circulation, especially due to virus imports. Germany´s history with two fundamental different vaccination strategies reveals that this transition phase is present irrespective of whether a country implemented compulsory vaccination or a recommendation to vaccinate. In order to keep this transition phase as short as possible, existing vaccination programs may need to be adjusted and have to be pursued with great consistency. The vaccination recommendation of adults implemented by the STIKO represents one essential part of this strategy and may be adopted by other (European) countries.

DEGS1 has been shown to be highly representative for the German adult population. For deviations of DEGS1 participants from the general German population was adjusted by weighting [19]. Still, bias may result from selective study participation of healthier individuals [59]. In addition, persons unable to provide written consent and those with significant language barriers were excluded from participation [19]. As a result, participants with migration background differed from the general population with respect to their age and education and therefore were only included in analyses if controlled for age and SES [60]. Individuals with low education were underrepresented within DEGS1. However, this effect was balanced by weighting [19]. About 3% of the German population migrates within Germany each year. Internal migration was especially observed by young adults from East to West Germany from 1991 to 2015 [61]. Although the share of population that migrates is too small to explain East-West differences within this study, bias caused by internal migration cannot be ruled out.

ELISA assays cannot distinguish between antibodies formed in response to natural infection or vaccination. Information on vaccination was collected within DEGS1 but was incomplete and error-prone due to self-reporting [25]. Countrywide vaccination policies were used for interpretation instead. Despite good timely correlation between alterations of vaccination policies and changes in seroprevalence, data interpretation has therefore to be considered with caution.

Low antibody titres in vaccinated, categorized as equivocal or negative by ELISA, can correlate with the presence of neutralizing antibodies (NRC MMR, unpublished data). Levels of neutralizing antibodies can be determined by PRNT which is, however, elaborate and costly and was therefore not conducted within DEGS1 [62]. Additionally, cell-derived immunity adds to the protection against the diseases which was also not detected by the approach used in this study [63]. Thus, consideration of seroprevalence only will result in an underestimation of population immunity [32,34,37].

The present data was collected before the recommendation on vaccination of adults born 1970 or later came into place and therefore allows the evaluation the effectiveness of this recommendation by follow-up studies. Time of data collection should be considered when relating data to the present situation in Germany.

## Conclusion

By identifying most vulnerable groups within the German adult population, the implementation of the STIKO recommendation on MMR vaccination of adults, as well as the implementation of the German Measles Protection Act, were reinforced. In addition, these data provide a base for further evaluation of these measures.

## Contributors

AM and CPM designed the study. NF and AM drafted the manuscript. NF, CPM and RK performed the statistical analyses. CPM, DMK, JK, OW and SaSa contributed to data interpretation and discussion. All authors edited and approved the manuscript. CPM and NF verified the underlying data.

## Data availability statement

The data set of the survey cannot be made publicly available because informed consent from study participants did not cover public deposition of data. However, the minimal data set underlying the findings presented in this manuscript is archived in the Research Data center at the Robert Koch Institute (RKI) and can be accessed by all interested researchers. On-site access to the data set is possible at the Secure Data center of the RKI's Research Data center. Requests should be submitted to fdz@rki.de.

## Declaration of interests

The authors have nothing to disclose.

## Appendix Supplementary materials

Image, application 1

Image, application 2
