# Supplementary material for: Seroprevalence of Measles-, Mumps-, and Rubella-specific antibodies in the German adult population – cross-sectional analysis of the German Health Interview and Examination Survey for Adults (DEGS1)
Source: Lancet Reg Health Eur. 2021 Jun 5;7:100128. doi: 10.1016/j.lanepe.2021.100128 (PMC8454806; doi:10.1016/j.lanepe.2021.100128)
Supplement: Supplementary file 1 [file mmc1.docx]

**Supplementary materials**

**Contents**

**Supplementary table S1.** Description of the population of analysis.

**Supplementary table S2**. Unadjusted odds ratios (OR) for the association between socio-demographic factors and negative measles, mumps, and rubella antibody titres in German adults born 1970 or later in 2008-2011.

**Supplementary figure S1.** Timeline of milestones of measles, mumps, and rubella vaccination in East, West and reunited Germany.

**Supplementary figure S2.** Flowchart of DEGS1 participant recruitment.

**Supplementary figure S3.** Measles vaccination and reported measles cases in East Germany (1962-1990).

**References**
