# Supplementary material for: Seroprevalence of Measles-, Mumps-, and Rubella-specific antibodies in the German adult population – cross-sectional analysis of the German Health Interview and Examination Survey for Adults (DEGS1)
Source: Lancet Reg Health Eur. 2021 Jun 5;7:100128. doi: 10.1016/j.lanepe.2021.100128 (PMC8454806; doi:10.1016/j.lanepe.2021.100128)
Supplement: Supplementary file 2 [file mmc2.pdf]

Supplementary materials

Contents

Supplementary table S1. Description of the population of analysis.

Supplementary table S2. Unadjusted odds ratios (OR) for the association between socio-demographic factors and negative measles, mumps, and rubella antibody titres in German adults born 1970 or later in 2008-2011.

Supplementary figure S1. Timeline of milestones of measles, mumps, and rubella vaccination in East, West and reunited Germany.

Supplementary figure S2. Flowchart of DEGS1 participant recruitment.

Supplementary figure S3. Measles vaccination and reported measles cases in East Germany (1962-1990).

References

Supplementary table S1. Description of the population of analysis.

	n	%
**Total**	7,115	100·00
**Gender**		
Men	3,410	47·93
Women	3,705	52·07
**Year of birth**		
1928-1934	305	4·29
1935-1939	722	10·15
1940-1944	788	11·08
1945-1949	637	8·95
1950-1954	652	9·16
1955-1959	730	10·26
1960-1964	718	10·09
1965-1969	599	8·42
1970-1974	468	6·58
1975-1979	383	5·38
1980-1984	434	6·1
1985-1993	679	9·54
**Region of residence**		
West	4,866	68·39
East	2,249	31·61
**Socio-economic status**		
low	1,133	15·92
middle	4,253	59·78
high	1,681	23·63
missing	48	0·67
**Migration background**		
None	5,869	82·49
One-sided	311	4·37
Two-sided	703	9·88
missing	232	3·26

Supplementary table S2. Unadjusted odds ratios (OR) for the association between socio-demographic factors and negative measles, mumps, and rubella antibody titres in German adults born 1970 or later in 2008-2011 (measles: n=1,845, mumps: n=1,570, rubella: n=1,723).

	**Measles**		**Mumps**		**Rubella**	
	**OR (95% CI)**	**p**	**OR (95% CI)**	**p**	**OR (95% CI)**	**p**
**Gender**		0·90		0·23		<0·01
Men	0·98 (0·68-1·40)		0·83 (0·61-1·13)		3·70 (2·13-6·41)	
Women	Referent		Referent		Referent	
**Year of birth**		0·55				<0·01
1970-1979	Referent		Referent	0·68	Referent	
1980-1993	1·11 (0·78-1·58)		0·94 (0·71-1·25)		1·96 (1·30-2·97)	
**Region of residence**		0·05		0·53		0·05
West	Referent		Referent		Referent	
East	1·42 (0·99-2·03)		0·90 (0·63-1·27)		0·58 (0·34-0·99)	
**Socio-economic status**		0·62		0·23		0·11
Low	Referent		Referent		Referent	
Middle	0·83 (0·54-1·26)		0·74 (0·52-1·06)		0·66 (0·39-1·09)	
High	0·79 (0·46-1·34)		0·90 (0·59-1·36)		0·50 (0·26-0·98)	
**Migration background**		n.a.[a]		n.a.[a]		n.a.[a]
None	n.a.[a]		n.a.[a]		n.a.[a]	
One-sided	n.a.[a]		n.a.[a]		n.a.[a]	
Two-sided	n.a.[a]		n.a.[a]		n.a.[a]	

[a]Are only to be analysed if stratified by age and socio-economic status.[1]

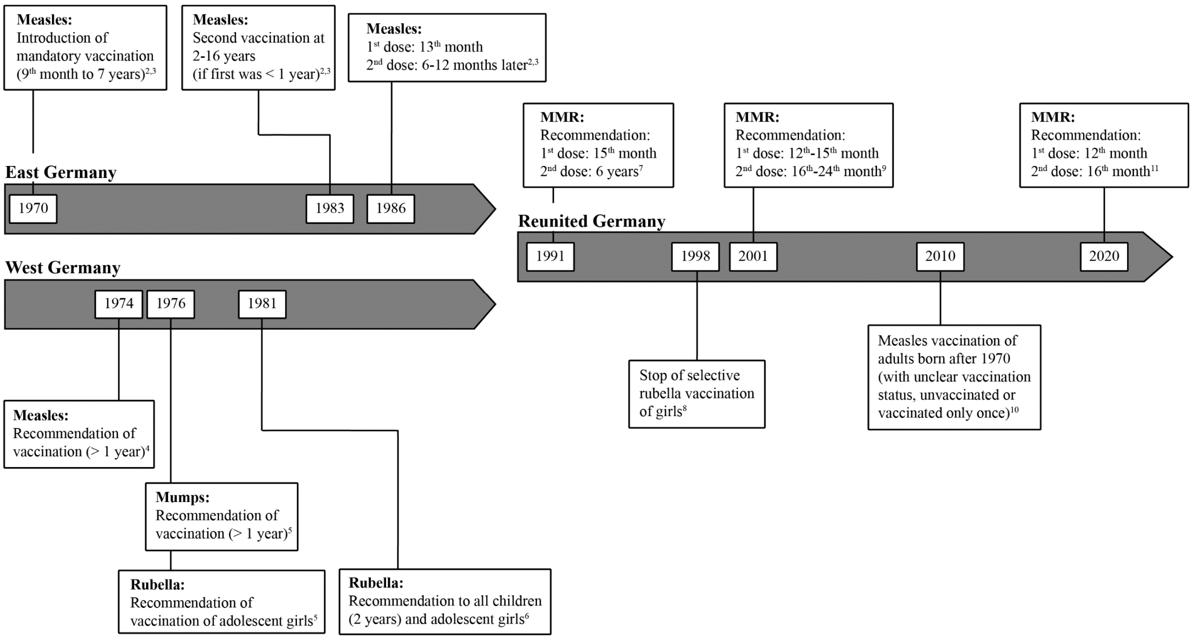

Supplementary figure S1. Timeline of milestones of measles, mumps, and rubella vaccination in East, West and reunited Germany.

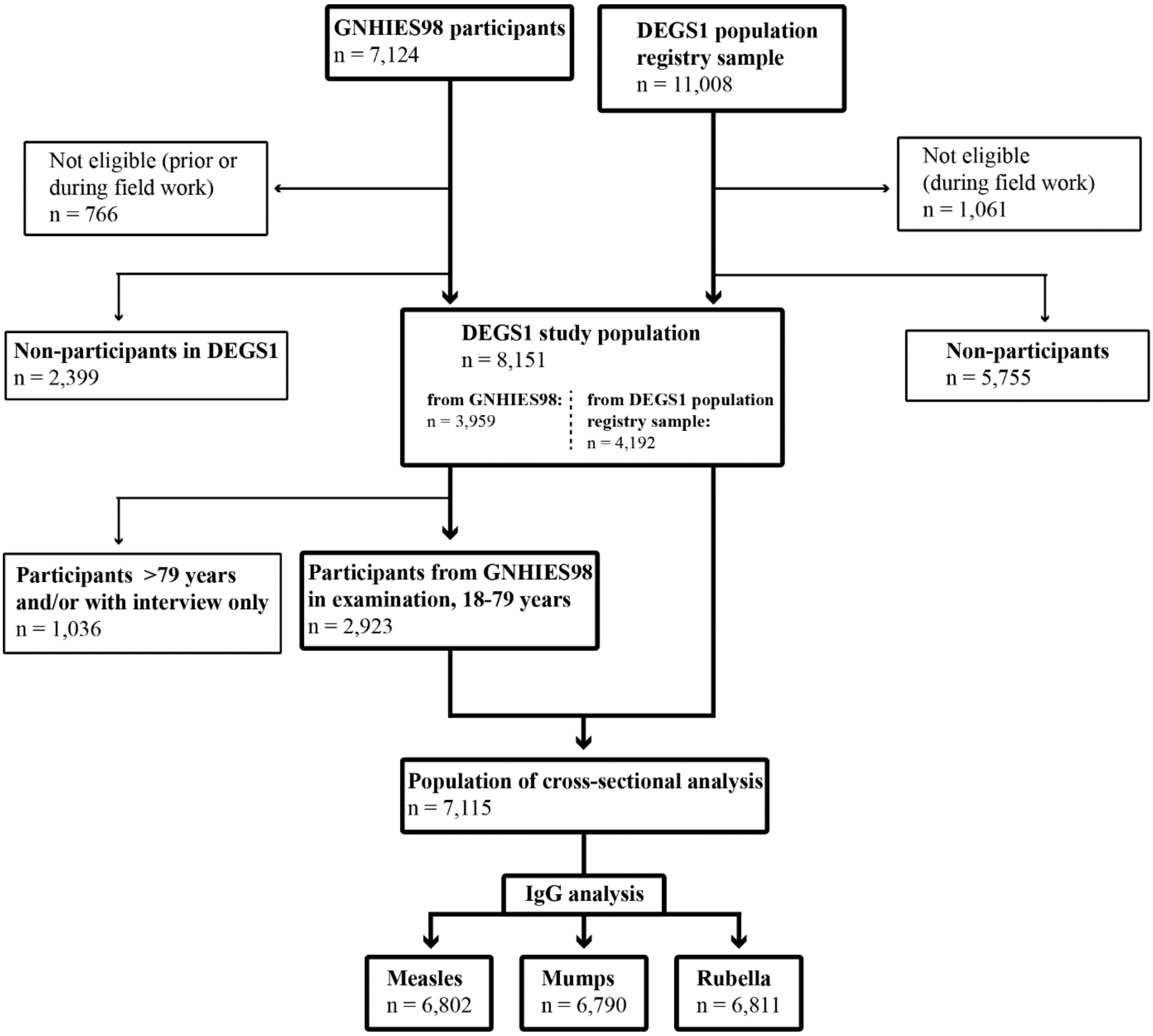

Supplementary figure S2. Flowchart of DEGS1 participant recruitment.

The study population of the German National Health Interview and Examination Survey for Adults (DEGS1) is composed out of former participants of the German Health Interview and Examination Survey (GNHIES98) and newly invited DEGS1 participants. For this, GNHIES98 participants who agreed to be re-contacted and were still contactable were re-invited to take part in DEGS1. To maintain representativeness, a population registry sample of n = 11,802 was newly invited to participate.[12] The resulting DEGS1 study population comprised 8,151 participants, of whom n = 3,959 were former GNHIES98 participants and n = 4,192 were newly recruited DEGS1 participants. Included in the cross-sectional analysis were participants aged 18-79 years with examination data available. This led to the exclusion of n = 1,036 of the former GNHIES participants either due to aging to >79 years and/or to participation in interviews only. Therefore, the population of analysis comprised 7,115 participants. IgG antibodies were determined of 6,802 (measles), 6,790 (mumps), and 6,811 (rubella) participants.

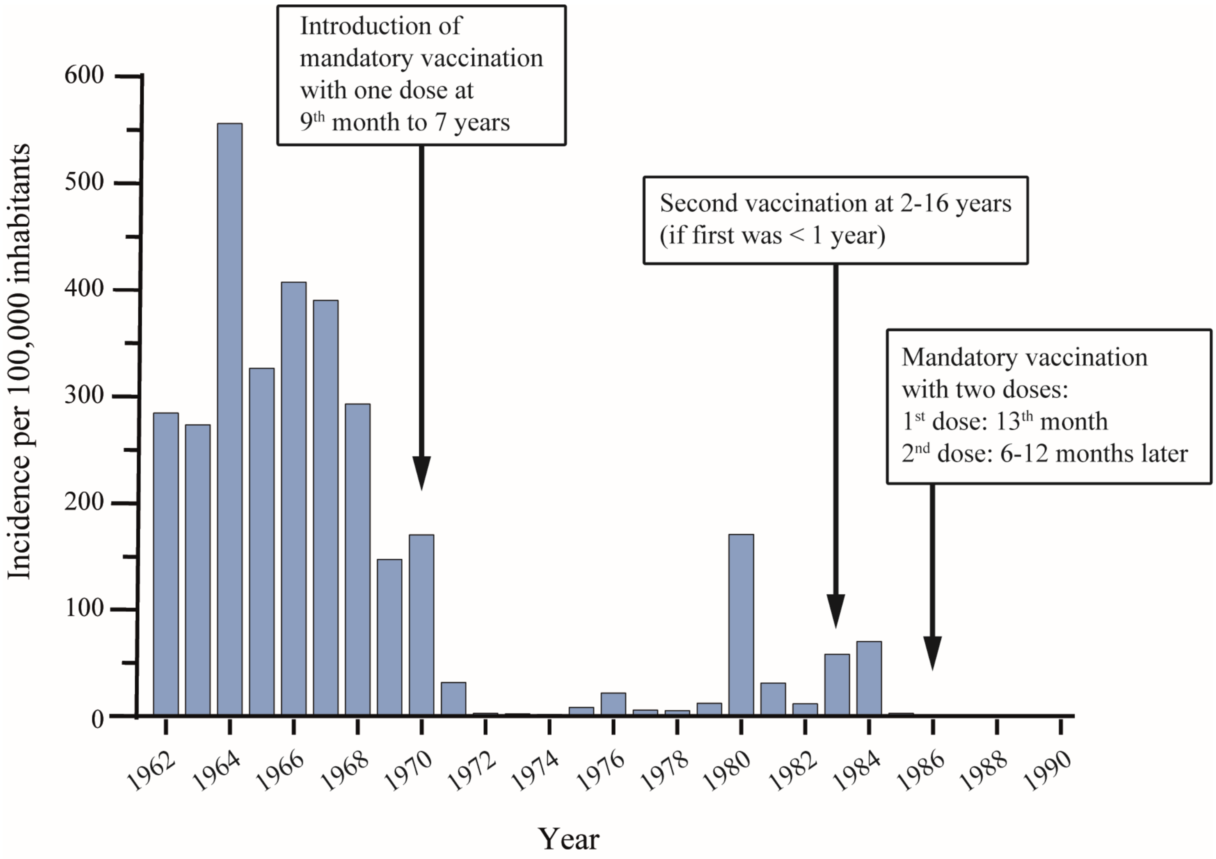

Supplementary figure S3. Measles vaccination and reported measles cases in East Germany (1962-1990).[13]

References

1. Sass A.C., Grune B., Brettschneider A.K., Rommel A., Razum O., Ellert U. [Participation of people with migration background in health surveys of the Robert Koch Institute]. *Bundesgesundheitsblatt Gesundheitsforschung Gesundheitsschutz* 2015; **58**(6): 533-42.
2. Dittmann S., Thilo W. Masern-Schutzimpfung. Vademekum für Impfärzte. Jena: Gustav Fischer Verlag; 1980: 105-12.
3. Dittmann S., Thilo W. Masern-Schutzimpfung. Vademekum für Impfärzte. Jena: Gustav Fischer Verlag; 1986: 93-102.
4. STIKO. STIKO-Empfehlung zur Masernschutzimpfung. Robert Koch-Institut, Infektionsepidemiologie; 1974.
5. STIKO. STIKO-Empfehlungen 1976. Robert Koch-Institut; 1976.
6. STIKO. Ergänzung der STIKO-Empfehlungen 1980. Robert Koch-Institut; 1981.
7. STIKO. STIKO-Empfehlungen 1991. Robert Koch-Institut; 1991.
8. STIKO. Impfempfehlungen der Ständigen Impfkommission (STIKO) am Robert Koch-Institut /Stand: März 1998. Robert Koch-Institute; 1998.
9. STIKO. Impfempfehlungen der Ständigen Impfkommission (STIKO) am Robert Koch-Institut /Stand: Juli 2001. Robert Koch-Institute; 2001.
10. STIKO. Impfempfehlungen der Ständigen Impfkommission (STIKO) am Robert Koch-Institut /Stand: Juli 2010. Robert Koch-Institute; 2010.
11. STIKO. Empfehlungen der Ständigen Impfkommission beim Robert Koch-Institut 2020/2021. Robert Koch-Institute; 2020.
12. Scheidt-Nave C., Kamtsiuris P., Gosswald A., et al. German health interview and examination survey for adults (DEGS) - design, objectives and implementation of the first data collection wave. *BMC Public Health* 2012; **12**: 730.
13. Wichmann O., Ultsch B. [Effectiveness, population-level effects, and heath economics of measles and rubella vaccination]. *Bundesgesundheitsblatt Gesundheitsforschung Gesundheitsschutz* 2013; **56**(9): 1260-9.
